# Transcriptomic and neurochemical analysis of the stellate ganglia in mice highlights sex differences

**DOI:** 10.1038/s41598-018-27306-3

**Published:** 2018-06-12

**Authors:** R. G. Bayles, A. Olivas, Q. Denfeld, W. R. Woodward, S. S. Fei, L. Gao, B. A. Habecker

**Affiliations:** 1 Department of Physiology and Pharmacology, Oregon Health & Science University, Portland, Oregon 97239 USA; 2 Biostatistics & Bioinformatics Core, Oregon National Primate Research Center, Oregon Health & Science University, Beaverton, OR 97006 USA

**Keywords:** Gene expression analysis, RNA sequencing, Transcriptomics, Autonomic nervous system, Cardiovascular biology

## Abstract

The stellate ganglia are the predominant source of sympathetic innervation to the heart. Remodeling of the nerves projecting to the heart has been observed in several cardiovascular diseases, however studies of adult stellate ganglia are limited. A profile of the baseline transcriptomic and neurochemical characteristics of the stellate ganglia in adult C57Bl6j mice, a common model for the study of cardiovascular diseases, may aid future investigations. We have generated a dataset of baseline measurements of mouse stellate ganglia using RNAseq, HPLC and mass spectrometry. Expression differences between male and female mice were identified. These differences included physiologically important genes for growth factors, receptors and ion channels. While the neurochemical profiles of male and female stellate ganglia were not different, minor differences in neurotransmitter content were identified in heart tissue.

## Introduction

The majority of sympathetic nerves projecting to the heart originate in the stellate ganglia, with innervation provided throughout the heart from both the left and right ganglion^1^. Coronary artery diseases, and their consequences such as acute myocardial infarction, cause alterations in cardiac neuronal function, which can contribute to the development of heart failure and cardiac arrhythmias^2^. Developing new therapeutic strategies to target peripheral sympathetic transmission will require a better understanding of the neural remodeling that occurs in disease, which is based on our characterization and understanding of the baseline physiology.

Transcriptomics are a powerful first step in the study of complex biological phenomena where the underlying regulatory networks are unknown. Recently, a number of important studies have begun to characterize the transcriptomic profile of the stellate ganglia in juvenile mice. These studies have focused on the developmental establishment of neuronal phenotype, including the dominant transcript isoforms characterizing sympathetic neurons in whole ganglia^3^, and the diversity of neuronal populations within the stellate ganglia as highlighted by single cell RNAseq^4^. With this study we aim to provide baseline measurements of the transcriptome in the adult stellate ganglia from C57Bl6j mice, which is a common mouse strain used in studies of cardiovascular disease. We have also quantified neurotransmitter levels in stellate ganglia and the heart. Given the number of studies demonstrating neuroanatomical, neurochemical and physiological sex differences in the brains of rodents^5^, and the different sex specific cardiovascular risk profiles in humans^6,7^, particular attention was paid to any underlying sex differences in this reference group.

## Methods

### Animals

Male and female (n = 6 each) C57Bl6j mice (age matched 16–19 weeks old) were obtained from Jackson Laboratories, and maintained on a 12 h: 12 h light/dark cycle with *ad libitum* access to food and water. Male mice were consistently heavier than female mice (Mean ± SEM: 22.8 ± 0.3 g for females and 29.0 ± 0.5 g for males, p < 0.001). All procedures were approved by the Oregon Health and Science University (OHSU) Institutional Animal Care and Use Committee and were in accordance with the Guide for the Care and Use of Laboratory Animals published by the National Academies Press (Ed 8). Mice were sedated with isoflurane, and the heart and sympathetic ganglia rapidly dissected. Left and right atria and ventricles were separated, as were the left and right stellate and superior cervical ganglia (SCG). Tissues collected for RNA were placed in RNAlater (Qiagen) and stored at −20 °C. Tissues from a second set of animals collected for neurochemical analyses were snap frozen and stored at −80 °C.

### RNAseq

The left stellate ganglion only was sequenced from each animal. RNA was extracted from ganglia using the RNAqueous Micro kit (Ambion) according to the manufacturer’s instructions. Briefly, single ganglia were homogenized using Wheaton micro tissue grinders (Thermo-Fisher) in lysis buffer (100 µL). The homogenate was added to ethanol (125 µL) in a separate tube and the protocol continued to elution in elution buffer (20 µL). Samples were stored at −80 °C. No DNAse treatment was performed to preserve yield. Each sample was assessed for RNA quality and integrity using a 2100 Bioanalyzer Instrument (Agilent). A single pooled sample of RNA comprised of both left and right SCG from 10 mice (5M, 5F) was prepared and sequenced as a reference library for calibration of future studies. This reference sample was not included in any analyses in this study, but is mentioned here due to its inclusion in the data normalization process. The SCG reference sample did not significantly affect the normalization of the 12 stellate ganglia samples (Supplementary information S1). Average RIN score for the RNA samples used was 6.7 for the stellate ganglia and 8.1 for the SCG pool. Sequencing libraries were prepared using the TruSeq Stranded Total RNA Library Prep Kit with RiboZero ribosomal RNA removal (Illumina), using 130 ng of RNA per sample. Sequencing was performed using an Illumina HiSeq 2500 through the Massively Parallel Sequencing Shared Resource at OHSU.

### Bioinformatics

The quality of the raw sequencing files were evaluated using FastQC^8^ combined with MultiQC^9^ (http://multiqc.info/). The files were imported into ONPRC’s DISCVR-Seq^10^, LabKey^11^ Server-based system, PRIMe-Seq. Trimmomatic^12^ was used to remove any remaining Illumina adapters. Reads were aligned to the Mus_musculus.GRCm38 genome in Ensembl along with its corresponding annotation, release 86. The program STAR (v020201)^13^ was used to align the reads to the genome. STAR has been shown to perform well compared to other RNA-seq aligners^14^. Two-pass mode was used. Since STAR utilizes the gene annotation file, it calculated the number of reads aligned to each gene. RNA-SeQC^15^ (v1.1.8.1) was utilized to ensure alignments were of sufficient quality. Raw RNAseq data consisted of an average of 60 M reads per sample, and 82% of reads mapped to unique transcripts and 11% mapped to multiple sites. Raw counts were determined for 48795 genes. Gene-level raw counts were filtered to remove genes with extremely low counts in many samples following the published guidelines^16^, specifically genes with extremely low counts were defined as those having no more than 0.2 counts per million (CPM) in a sample, corresponding to a count of 8–9 in the smallest library. Genes with extreme low counts in at least 7 out of 12 samples were filtered out to ensure a minimum of expression in a single sex group. 19638 genes were retained after extreme low count filtering.

Gene-level differential expression analysis was performed in open source software R^17^. Gene-level raw counts were normalized using the trimmed mean of M-values method (TMM)^18^, and transformed to log-counts per million with associated observational precision weights using voom^19^ method. Gene-wise linear models comparing the sexes were employed for differential expression analyses using limma with empirical Bayes moderation^20^ and false discovery rate (FDR) adjustment^21^.

The following metrics were generated. log2FC is the fold change of gene expression in log2 scale (fold difference). For example, log2FC of 1 corresponds to fold difference of 2 (up-regulated) and log2FC of −1 corresponds to fold difference of 0.5 (down-regulated). pvalue is the empirical Bayes moderated p-value for that particular contrast. FDR is the false discovery rate adjusted p-value.

### Real-time PCR

Select expression differences were confirmed using additional RNA from the left stellate ganglia that were sequenced. In additional follow-up analyses, gene expression was also quantified in left SCG from additional age-matched animals. Reverse transcription was performed using the iScript reverse transcription supermix (BioRad) according to the manufacturer’s instructions.

The following TaqMan Gene Expression Assays (Thermo-Fisher) were used for real-time PCR analysis: *Gapdh*, Mm99999915_g1; *Th*, Mm00447557_m1; *Kcna1*, Mm00439977_s1; *Kcna2*, Mm01546131_g1; *Kcnn4*, Mm00464586_m1; *Tcfl5*, Mm00626495_m1; *Ntrk3*, Mm00456222_m1; *Slc18a1*, Mm00461868_m1; *Ntng1*, Mm00453144_m1; Ret, Mm00436304_m1.

### HPLC analysis of Norepinephrine (NE) and mass spectrometry analysis of Acetylcholine (ACh)

Frozen tissue was used for neurotransmitter analysis. All tissue was homogenized in perchloric acid (300 µl, 0.1 M) containing dihydroxybenzylamine (1.0 µM) internal standard. Ganglia were homogenized without weighing. Atria were weighed and added directly to the homogenization buffer. Ventricles were pulverized using a mortar and pestle on dry ice, prior to weighing and homogenization of a small amount of tissue. After homogenization, all samples were centrifuged (13000 g for 5 min). Catecholamines were purified from an aliquot (100 µL) of the supernatant by alumina adsorption. A second aliquot (100 µL) was filtered at 4 °C for ACh quantification. NE, the NE metabolite dihydroxyphenylglycol (DHPG), dopamine (DA) and the dopamine metabolite 3,4-dihydroxyphenylacetic acid (DOPAC) levels were measured by HPLC with electrochemical detection and ACh was quantified by mass spectrometry as described previously^22^. Detection limits were ~0.05 pmol with recoveries from the alumina extraction >60%.

### Statistics

Graphing and statistical analysis were performed using Microsoft Excel and GraphPad Prism v6. Binary comparisons between males and females for individual genes (qPCR) were performed using Student’s t-tests.

## Results

**Figure 1:**
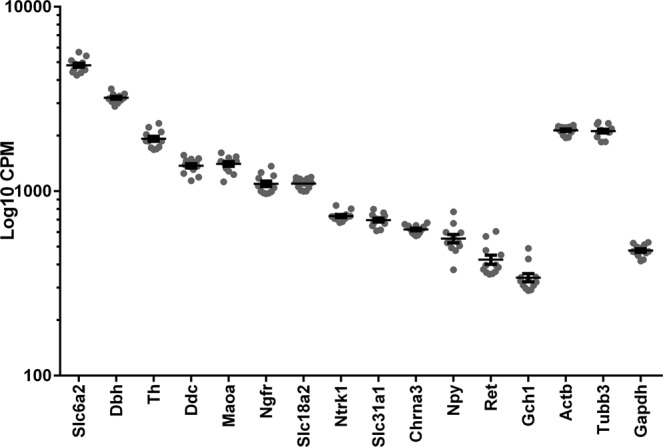
Expression of sympathetic markers: Left stellate ganglion expression levels of genes in both male and female mice using normalized Log_10_CPM values (Counts Per Million); Mean ± SEM. Select genes important in sympathetic function are presented in addition to 3 commonly used housekeeping genes (*Actb*, *Tubb3* and *Gapdh*) for reference. Genes specific to sympathetic neurons are among the most highly expressed in the stellate.

The genes most closely associated with sympathetic neuron function were among the most highly expressed genes in both male and female left stellate ganglia (Fig. 1). These include the genes encoding proteins required for NE synthesis (*Th*, tyrosine hydroxylase; *Ddc*, DOPA decarboxylase; *Gch1*, GTP cyclohydrolase 1; *Dbh*, dopamine beta hydroxylase), packaging into vesicles (*Slc18a2*, vesicular monoamine transporter 2) and removal from the neuroeffector junction (*Slc6a2*, norepinephrine transporter), catabolism (*Maoa*, monoamine oxidase A) as well as the sympathetic co-transmitter Neuropeptide Y (*Npy*). Sympathetic neurons rely on Nerve Growth Factor (NGF) for survival, and genes encoding the two receptors for NGF – *Ngfr* (p75 neurotrophin receptor) and *Ntrk1* (TrkA tyrosine kinase receptor) – were likewise expressed abundantly in both sexes. The 100 most highly expressed genes were almost identical between sexes (Supplementary data; Sheet 2). The relative abundance of genes encoding sympathetic markers matched the patterns of expression reported in previous RNAseq analyses carried out in younger mice whose sympathetic neurons were heterozygous for the transcription factors *Hand2* or *Gata3* (Graphed in Supplementary data; Sheet 4)^3,23^. The expression levels of other gene classes relevant to sympathetic nerve function in our dataset were also consistent with the levels reported in the previous study (Supplementary information; Figures 3–9).

Given the uniform high expression of genes related to neurotransmission in ganglia from both sexes, we did not expect to see differences in neurotransmitter levels between males and females. To determine if that was the case, we quantified NE and ACh in hearts and sympathetic ganglia from male and female mice. Cardiac neurotransmitter levels were generally similar between the sexes, except for the right atrium where females had significantly higher levels of ACh (Table 1). There was also a significantly higher level of DHPG, the primary neuronal metabolite of NE, in the right atrium of female mice when compared to both the male right atrium and the female left atrium. The DHPG to NE ratio was significantly higher in the atria compared to the ventricles in both sexes. No significant differences in neurotransmitter or metabolite levels were observed in stellate ganglia between male and female mice (Table 2). DHPG was not detected in the stellate ganglia. Consistent with previous findings in rats we found readily detectable levels of dopamine and its primary metabolite DOPAC in stellates from both sexes^24^, but not in the heart.

**Table 1:** Cardiac neurochemistry.

		NE (pmol/mg)	DHPG (pmol/mg)	ACh (pmol/mg)
**Atria**				
Right	Male	4.8 ± 2.3	3.1 ± 0.9	5.7 ± 1.7
	Female	6.9 ± 2.1	6.1 ± 1.8*	9.3 ± 2.9*
Left	Male	3.8 ± 1.3	3.0 ± 1.6	4.8 ± 1.8
	Female	5.9 ± 3.2	3.2 ± 0.9	6.1 ± 2.8
**Ventricle**				
Right	Male	3.9 ± 0.6	0.5 ± 0.1	1.5 ± 0.2
	Female	3.9 ± 1.2	0.4 ± 0.1	1.8 ± 0.3
Left	Male	3.2 ± 0.3	0.6 ± 0.1	0.8 ± 0.2
	Female	2.9 ± 0.2	0.5 ± 0.1	0.9 ± 0.1

Chamber-specific levels of NE; Norepinephrine, DHPG; Dihydroxyphenylglycol, and ACh; Acetylcholine. Female mice had higher levels of DHPG and ACh in the right atrium of the heart than males. No differences in neurotransmitter levels were detected in the ventricles. Mean ± SD, n = 4–6; *p < 0.05 vs male.

**Table 2:** Stellate ganglion neurochemistry.

		NE	ACh	Dopamine	DOPAC
Left	Male	61.1 ± 3.7	19.7 ± 4.3	6.7 ± 1.5	6.4 ± 3.2
	Female	59.0 ± 9.9	18.0 ± 3.4	6.7 ± 1.9	7.1 ± 1.9
Right	Male	54.7 ± 7.5	20.6 ± 2.9	7.2 ± 1.4	7.2 ± 3.9
	Female	61.6 ± 9.2	18.4 ± 2.7	7.2 ± 1.5	7.8 ± 1.5

No differences in neurotransmitter levels were detected in the stellate ganglia between the left and right side, or between the sexes. Units; pmol/ganglion, Mean ± SD, n = 6. DOPAC; Dihydroxyphenylacetic acid.

**Figure 2:**
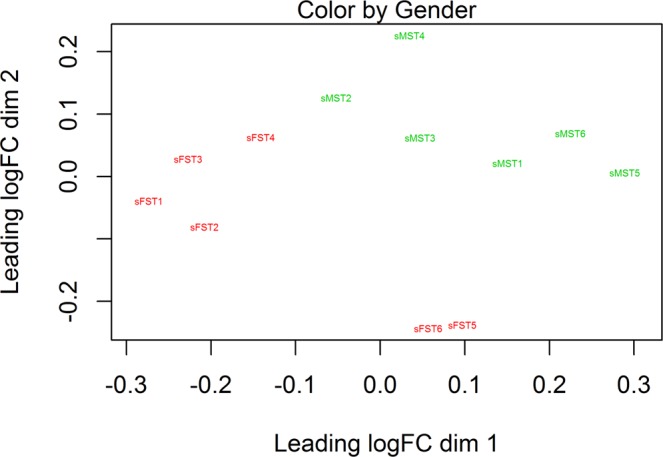
Multidimensional Scaling Plot of log-CPM values: MDS plot over 2 dimensions highlighting clear separation of sexes. MST, Male Stellate (Green); FST, Female Stellate (Red). The scale indicates log2 fold difference in expression (Normalised CPM, counts per million) across each dimension.

Despite the many similarities in gene expression between male and female stellate ganglia, there were also many genes that were differentially expressed. Separation of the sexes was observed during quality control of the dataset which included multidimensional scaling (MDS) analysis to determine how the expression profiles of each sample compared to all others (Fig. 2). Subsequent regression analysis determined that at a FDR adjusted p-value < 0.05, 12 genes had greater expression in females, and 9 greater in males. At a FDR adjusted p-value < 0.2, 70 genes had greater expression in females, and 34 greater in males. Genes significantly different between the sexes at a FDR adjusted p-value < 0.05 are listed in Table 3. There was no strong evidence of considerable pathway enrichment among the differentially expressed genes using Panther v13^25^ and Ingenuity Pathway Analysis (Qiagen) (data not shown).

**Table 3:** Genes that are differentially expressed between male and female (FDRp < 0.05).

Gene ID	Chromosome	Fold difference	p-value	FDR adjusted p-value
*Uty*	Y	10.56	8.98E-17	1.76E-12
*Gm27520*	X	−7.41	1.84E-16	1.81E-12
*Gm29650*	Y	7.33	3.69E-16	2.42E-12
*Kdm5d*	Y	10.49	2.42E-15	1.19E-11
*Eif2s3y*	Y	11.16	7.14E-15	2.80E-11
*Xist*	X	12.06	1.98E-14	5.56E-11
*Ddx3y*	Y	−9.49	1.84E-14	5.56E-11
*Tsix*	X	−8.30	3.88E-11	9.52E-08
*Kdm6a*	X	−0.79	1.50E-10	3.26E-07
*Eif2s3x*	X	−0.57	5.02E-08	9.85E-05
*1810041L15Rik*	15	−0.38	8.14E-07	0.0015
*Kdm5c*	X	−0.46	2.10E-06	0.0034
***Ntrk3***	7	−0.36	6.96E-06	0.0100
*Mitf*	6	−0.80	7.16E-06	0.0100
*Eps8l1*	7	−1.07	8.83E-06	0.0116
*5530601H04Rik*	X	0.64	1.05E-05	0.0122
***Tcfl5***	2	−0.45	1.04E-05	0.0122
*Slco3a1*	7	0.34	2.31E-05	0.0252
*Efhd1*	1	−0.77	3.38E-05	0.0349
***Ntng1***	3	0.34	4.51E-05	0.0442
*Acat2*	17	0.30	4.88E-05	0.0456

Most differentially expressed genes sorted by FDRp-value (False discovery rate adjusted p-value comparing males and females). Fold difference; Difference in expression between males and females, where a positive number represents more expression in males compared to females, and a negative number represents less expression in males compared to females. p-value; uncorrected p-value comparing males and females. The most differentially expressed genes were located on the sex chromosomes, however autosomal genes were also significantly different between the sexes. Bold; Genes of interest confirmed by qPCR.

**Figure 3:**
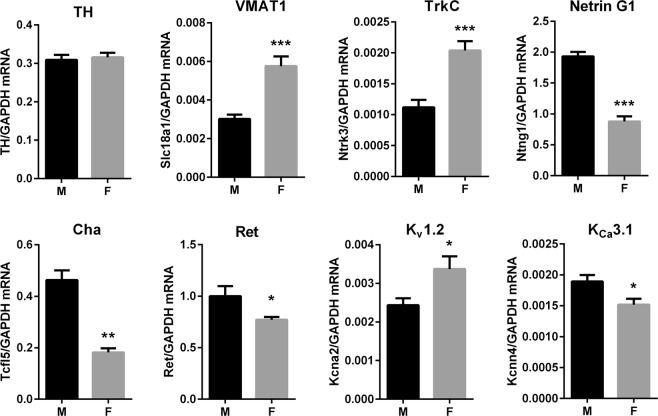
qPCR quantification of selected genes of interest in the stellate ganglion: Relative gene expression levels expressed as a ratio to *Gapdh* expression in left stellate ganglion (Mean ± SEM, n = 6 M vs 6 F). There was strong agreement between RNAseq and qPCR results for select genes of interest. *p < 0.05, **p < 0.01, ***p < 0.001.

As expected, many of the differentially expressed genes are present on the X or Y chromosome. The top 8 most statistically significantly different genes also clearly had the largest fold difference, representing sex chromosome specific genes including chromatin modifiers involved in silencing of the inactive X chromosome. However, several genes that exhibited differential expression were surprising, including the genes encoding TrkC, netrin G1, and a Chagas’ associated protein (Table 3, bold). These genes, and additional genes of known clinical interest with FDRp < 0.2 were selected for quantification by real-time PCR using additional RNA from the same samples that had been sequenced. Despite small fold differences detected by RNAseq, expression by qPCR was highly consistent with RNAseq data. While *Th* expression relative to *Gapdh* served as an effective negative control for sex differences, there was significantly more expression of *Slc18a1* (VMAT1), *Ntrk3* (TrkC), and *Kcna2* (K_v_1.2) in the stellate ganglia of female mice, while there was significantly more expression of *Ntng1* (Netrin G1), *Tcfl5* (Cha), *Ret* (Ret) and *Kcnn4* (K_Ca_3.1) in male mice (Fig. 3). The fold difference and p-values for all genes are available in the Supplementary data; Sheet 1.

**Figure 4:**
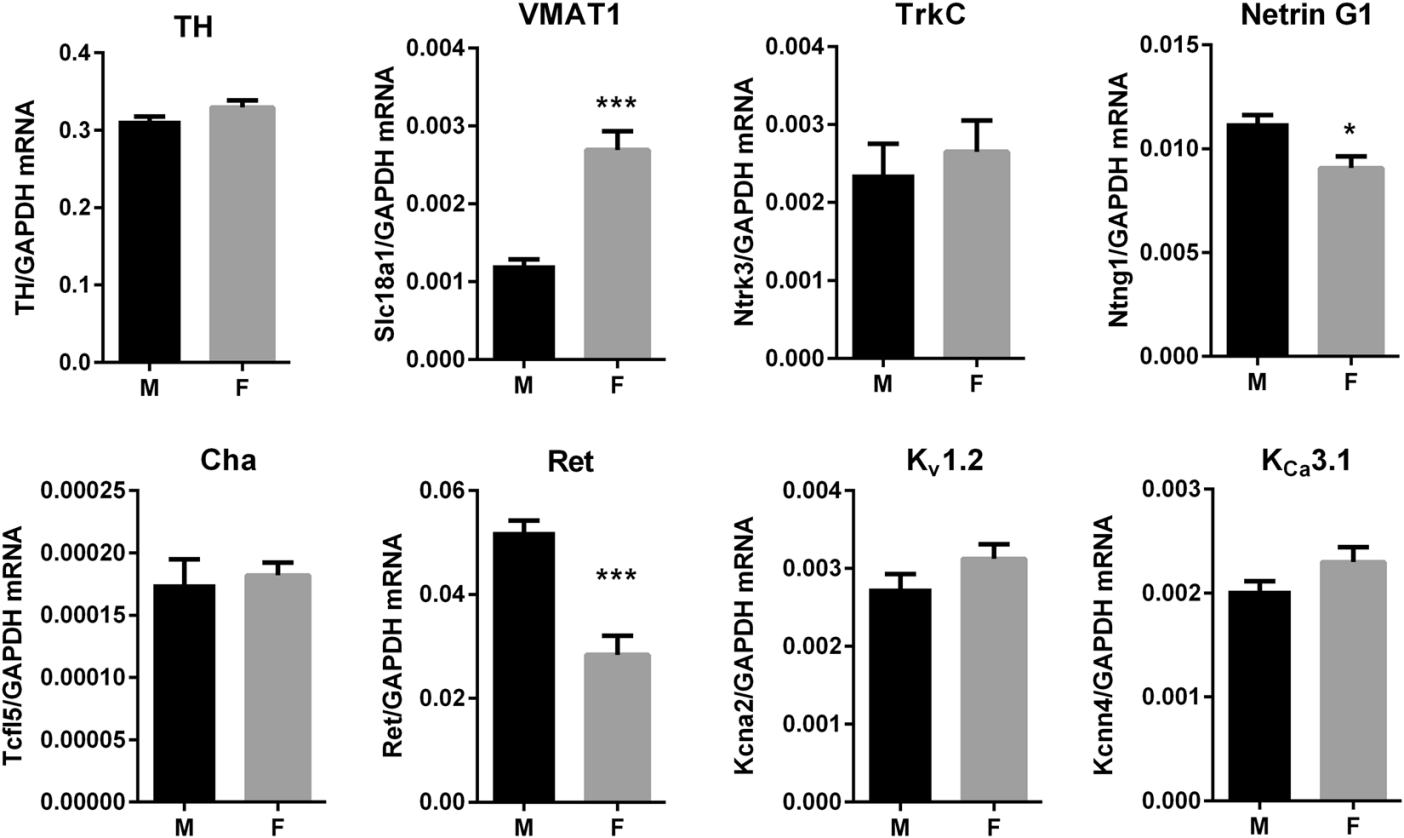
qPCR quantification of selected genes of interest in the SCG: Gene expression was assessed in the SCG as an alternative sympathetic ganglion. Relative gene expression levels expressed as a ratio to *Gapdh* expression in left SCG (Mean ± SEM, n = 6 M vs 4 F, n = 3 F for *Ret*). The gene expression levels of *Ntng1, Ret* and *Tcfl5* were much lower in the SCG compared to the stellate. The sex differences for *Slc18a1, Ret* and *Ntng1* only were present in both the stellate and SCG. *p < 0.05, **p < 0.01, ***p < 0.001.

It was surprising that genes encoding proteins such as ion channels were expressed at different levels in male and female stellate ganglia, and we wondered if those differences were specific to stellate ganglia or if they were general differences across sympathetic ganglia. Therefore, we quantified the same genes in SCG to determine if these differences were unique to stellate ganglia, or were present in another sympathetic ganglion (Fig. 4). Interestingly, the only genes that exhibited differential expression in male vs female SCG were the genes encoding VMAT1, Ret and Netrin G1. Most of the genes that were differentially expressed in stellate ganglia were expressed to a similar degree in SCG from male and female mice, including the genes encoding potassium channels. Expression levels were similar in both the stellate ganglion and SCG for most genes, however the genes encoding Netrin G1, Ret and Cha had much lower expression in the SCG relative to the stellate, (by approximately 2000-fold for Cha).

With the recent work of Furlan *et al*.^4^, establishing subtypes of peripheral sympathetic neurons based on distinct transcriptomic profiles, it was possible to investigate whether there might be any differences in these neuronal subpopulations between the sexes. For example, Ret is an important developmental growth factor receptor that was found to be a postnatal marker of cholinergic type neurons. Investigating the subpopulation specific genes from single cell analysis^4^ in our dataset, the average normalized gene counts of at least 6 genes were significantly different between the sexes, including *Ret* and genes related to the NA4 and NA5 subtypes of sympathetic neurons as defined by Furlan *et al*. (Supplementary information; Figure S2, Supplementary data; Sheet 5).

## Discussion

In the present study we have generated a reference transcriptome of the stellate ganglia in adult C57Bl6j mice with associated tissue neurochemistry. Overall, the sequencing profiles were highly consistent between all animals, however significant differences were detected between the sexes both in terms of gene expression and atrial neurotransmitter levels. The most abundant genes within all ganglia related to neurotransmission, and these genes were present at identical levels in male and female ganglia. Several genes that were differentially expressed in stellate ganglia were expressed at similar levels in male and female SCG, raising the possibility that the sex differences in gene expression might correlate with innervation of particular target tissues.

We were interested to see if differences in tissue neurotransmitter content between the sexes could be detected when comparing both the stellate ganglia themselves, and the tissues they project to. We did not detect any differences between sexes in neurotransmitter content of the stellate ganglia (Table 2). Concentrations were consistent across animals and may serve as a reference. We did not detect DHPG in the stellate, likely due to the majority of NE metabolism occurring following release, remote to the cell body. The presence of dopamine and its major metabolite DOPAC has previously been reported for sympathetic ganglia, and is often attributed to the presence of SIF cells^26^. In contrast, we did not detect dopamine or DOPAC in the heart (Table 1). An increased acetylcholine content was observed in the right atrium in female mice. While there was no significant difference in NE levels in the right atria, there were significantly higher levels of the metabolite DHPG in female mice, perhaps suggesting increased NE turnover. If the left and right atria were combined, there was a significantly higher concentration of NE, DHPG and ACh in the atria of female mice (Data not shown). These differences could not be explained by the expression of genes involved in neurotransmitter transport and metabolism, which were consistent across sexes.

Although the genes associated with noradrenergic function were expressed at similar levels in male and female ganglia, sex differences were observed in potentially important functional classes of genes. Results of qPCR for select genes were in strong agreement with RNAseq results, despite the small fold differences observed. These included genes for the TrkC receptor, the vesicular monoamine transporter VMAT1, the neuronal growth factor Netrin G1, the growth factor receptor Ret, and ion channels including K_Ca_3.1 and K_V_1.2 (Fig. 3). The mRNA levels of the genes for these proteins have been shown previously to correlate well with protein levels and are functionally relevant. For example, *Ntrk3* mRNA expression levels in breast cancer correlate well with TrkC protein levels and function, although other regulatory mechanisms are also important^27^. *Ntng1* mRNA levels are important in regulating developmental expression of Netrin G1 protein, an axonal guidance cue with a potential role in psychiatric disease^28,29^. Good agreement has been previously observed between *Slc18a1* mRNA and VMAT1 protein expression levels^30^, although VMAT1 is typically non-neuronal, and expression detected in sympathetic ganglia may be limited to SIF cells^31^.

Several genes identified by Furlan *et al*.^4^ as defining specific subsets of neurons were significantly different between the sexes, including the genes encoding VMAT1 (*Slc18a1*), the NPY receptor 2 (*Npy2r*), the growth factor receptor *Ret*, and ectoderm-neural cortex protein 1 (*ENC1*). (See Supplementary information; Figure S2 and Supplementary data; Sheet 5). Many of the genes that were differentially expressed in our RNAseq data set are associated with the NA4/5 and cholinergic neuron subtypes defined by Furlan *et al*. That study provided compelling evidence that target interactions were crucial in driving the expression of subtype-specific genes. NPY, which is present in the sympathetic neurons innervating the heart, is associated with neuronal subtypes NA2 and NA3 suggesting that many of the sex differences identified in our study arise from neurons projecting to non-cardiac targets. This is an important area that requires further investigation.

Analysis in the SCG showed that while some sex differences were confirmed in both the stellate and SCG, others were specific to the stellate. The expression levels of some genes were also significantly different between the stellate and SCG, likely related to differences in the target tissues that these ganglia innervate (Fig. 4). The expression of *Slc18a1* (VMAT1) and *Ntrk3* (TrkC) was similar in the stellate and SCG. However, there was a sex difference for *Slc18a1* in both the stellate ganglia and SCG as opposed to *Ntrk3* which was not different in the SCG. The levels of *Ntng1* (Netrin G1) and *Ret* were much lower in the SCG compared to the stellate, but with a consistent difference between the sexes.

Antibodies to epitopes from Cha, the transcription factor encoded by the gene *Tcfl5* (see Fig. 3), are highly specific markers of Chagas’ Disease, that are present in the sera of patients infected by *Trypanosoma cruzi*^32^. The levels of *Tcfl5* were approximately 1000-fold lower in the SCG compared to the stellate ganglia, with a difference between the sexes only observed in the stellate ganglia (Fig. 4). This may imply a particular importance of Cha expression in relation to the innervation of the heart and other cardiovascular targets, given that the majority of the cardiac innervation originates in the stellate ganglia^1^. Sympathetic ganglionitis has been observed in Chagas’ Disease^33^, and cardiac denervation, arrhythmia and associated sudden cardiac death are a common outcome in these patients^34^. There is ongoing debate as to whether autoantibodies may play a functional role in the disease, but immune infiltration of the ganglia alone could affect nerve activity and phenotype. Recent clinical studies suggest that inflammation and immune cell infiltration of the stellate ganglia is a common feature in patients with severe arrhythmias^35,36^. Increased cardiac morbidity and mortality is observed in males with Chagas’ Disease^37^. Given that we saw more than twice as much *Tcfl5* mRNA expression in the stellate ganglia of male mice, it would be interesting to know if the inflammatory state of the stellate ganglia is different between the sexes in Chagas’ Disease.

We saw an altered expression profile of a number of ion channels between the sexes, which was more pronounced in the stellate ganglia than the SCG. Such differences could potentially translate directly to differences in risk of arrhythmia between males and females, given the importance of transcriptional regulation on ion channel function. *Kcna2* mRNA has been shown to be differentially expressed in response to sex hormones in fish, directly effecting function of electrocytes^38^. A series of papers by Tao *et al*. has established the importance of *Kcna2* mRNA regulation in neuropathic pain, where transcriptional repression following nerve injury leads to changes in neuronal excitability^39–41^. Further investigation is required to determine whether the resting membrane potential or excitability of the cardiac sympathetic nerves is different at baseline between males and females.

It is commonly understood that there are going to be differences between males and females in animal studies related to hormonal differences and fundamental sex physiology. It is easy to overlook gender differences as obvious, and perhaps common to work only with male animals in disease models. Sex differences are clinically important, however, and not just an experimental inconvenience. As researchers increasingly recognize the importance of studying both male and female animals in disease models, it becomes increasingly clear that a sound understanding of the baseline physiology needs to be established in both sexes, in line with ongoing efforts of the NIH^42^. Previous sequencing studies of sympathetic ganglia have generally used male animals only, or a combination of males and females. Our data suggests that careful attention should be paid to the sex of animals in such studies. In some cases, retrospective analyses may be possible. For example in the case of the single cell sequencing that was reported by Furlan *et al*.^4^, retrospective sex typing of the single cells based on sex chromosome specific transcripts identified in our study (Table 2) may be possible.

Genes associated with the sex chromosomes were expected to be differentially expressed between males and females, and since sex is the most obvious variable in this animal cohort, these differences stand out (Fig. 2). However the discovery of differences in physiologically important autosomal genes such as those related to neuronal growth factors, neurotransmitter metabolism and ion channels was perhaps unexpected. While it is beyond the scope of this study to investigate how gonadal hormones may influence differences highlighted in this work, it is important to note that the first gene to be clearly linked with non-hormonal sex differences in mammals, *SRY*, was shown to effect the expression of TH in the brain^43,44^. In our study, we found no difference in TH expression between the sexes, and *SRY* expression was not detected in the stellate ganglia. It is unclear if the gene expression differences detected in this study translate to a difference in the baseline electrophysiology of sympathetic nerves between male and female mice, so that will be an important area for future investigation.

## Electronic supplementary material

Supplementary Information

Supplementary Dataset

## Data Availability

The datasets generated during and/or analysed during the current study are available in the NCBI Sequence Read Archive (SRA **SRP130366**), or as supplementary material.
