# Publisher Correction: Transcriptomic and neurochemical analysis of the stellate ganglia in mice highlights sex differences

**DOI:** 10.1038/s41598-019-45211-1

**Published:** 2019-06-26

**Authors:** R. G. Bayles, A. Olivas, Q. Denfeld, W. R. Woodward, S. S. Fei, L. Gao, B. A. Habecker

**Affiliations:** 1 Department of Physiology and Pharmacology, Oregon Health & Science University, Portland, Oregon 97239 USA; 2 Biostatistics & Bioinformatics Core, Oregon National Primate Research Center, Oregon Health & Science University, Beaverton, OR 97006 USA

Correction to: *Scientific Reports* 10.1038/s41598-018-27306-3, published online 12 June 2018

Two supplementary files containing Figures S1 - S9 and the supplementary datasets were omitted from the original version of this Article.

These errors have been corrected in the HTML version of the Article; the PDF version was correct at time of publication.

