# Supplementary material for: Transcriptomic and neurochemical analysis of the stellate ganglia in mice highlights sex differences
Source: Sci Rep. 2018 Jun 12;8:8963. doi: 10.1038/s41598-018-27306-3 (PMC5997635; doi:10.1038/s41598-018-27306-3)
Supplement: Supplementary file 1 — Supplementary Information [file 41598_2018_27306_MOESM1_ESM.docx]

**Supplementary Information**

**Transcriptomic and neurochemical analysis of the stellate ganglia in mice highlights sex differences**

Bayles RG, Olivas A, Denfeld Q, Woodward WR, Fei SS, Gao L, Habecker BA


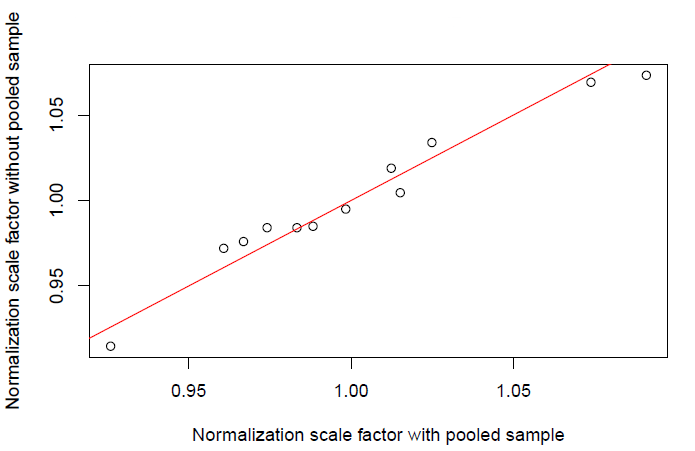


**Figure S1: Comparison of scale factor with or without the pooled SCG sample**

With the goal of having a reference sample for calibration of future experiments, the effect of this sample on the normalization of the dataset was determined. Inclusion of the sample did not significantly affect the normalization of the dataset, therefore the SCG pooled reference sample was included for the normalization process.


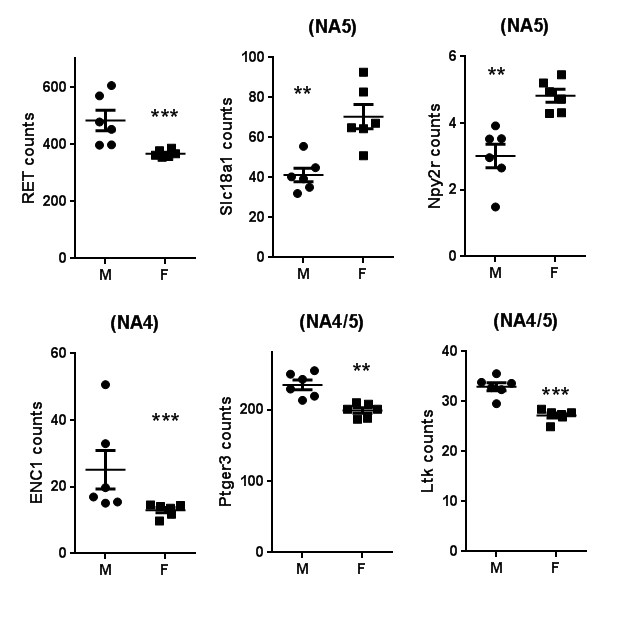


**Figure S2: Neuronal population subtype gene expression differences**

**A)** Normalized gene count values comparing RNAseq transcripts between male and female mice (Stellate ganglia RNAseq data n=6). NA4/5 refers to the neuronal population subtypes as defined by **Furlan et al. (2016)**.

qPCR confirmed differential *Ret* expression in stellate and superior cervical ganglia (**Figures 3&4**).

Student’s *t*-test*p<0.05, **p<0.01, ***p<0.001.

**Figures S3-9 display the relative gene count numbers of different isoforms of some important classes of neuronal genes in the left stellate ganglia.**


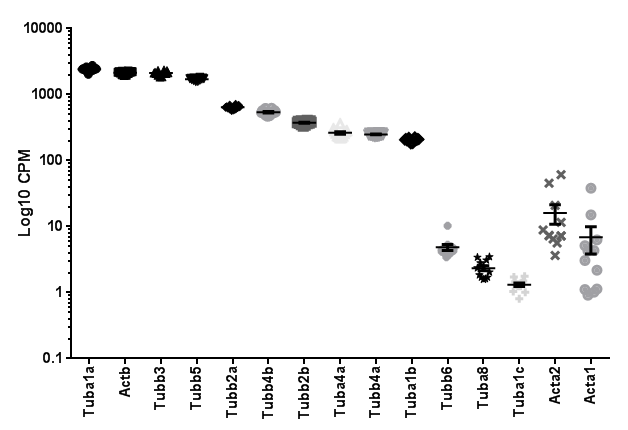


**Figure S3: Actin and Tubulin gene isoform expression in the stellate**

Average expression across all samples ±SEM (n=12) expressed as Log10 Counts per Million (CPM)


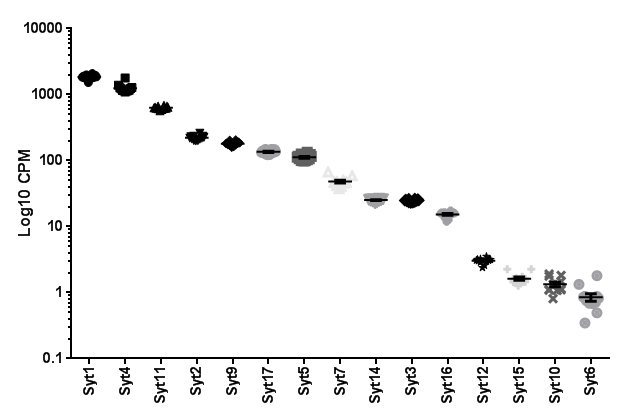


**Figure S4: Synaptotagmin gene isoform expression in the stellate**

Average expression across all samples ±SEM (n=12) expressed as Log10 Counts per Million (CPM)


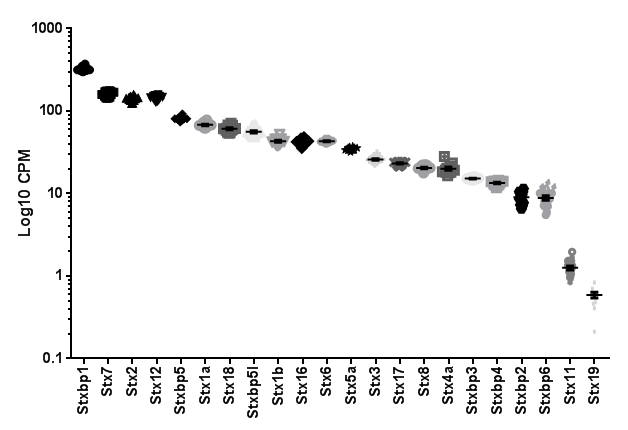


**Figure S5: Syntaxin gene isoform expression in the stellate**

Average expression across all samples ±SEM (n=12) expressed as Log10 Counts per Million (CPM)


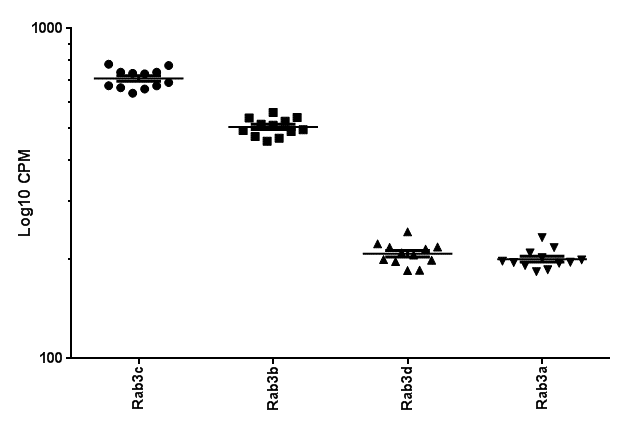


**Figure S6: Rab3 gene isoform expression in the stellate**

Average expression across all samples ±SEM (n=12) expressed as Log10 Counts per Million (CPM)


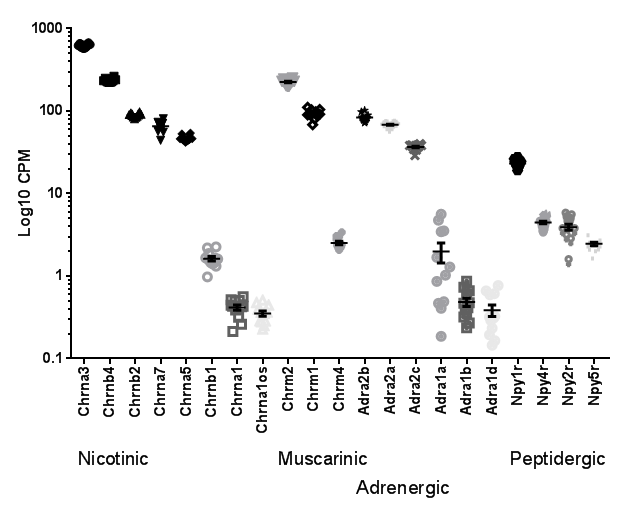


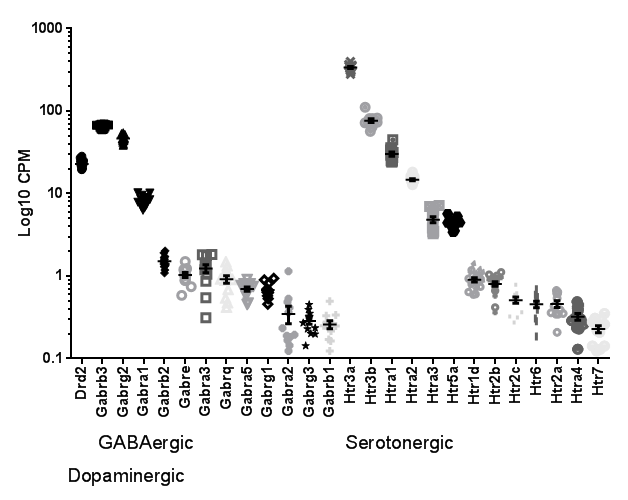


**Figure S7: Receptor gene isoform expression in the stellate**

Average expression across all samples ±SEM (n=12) expressed as Log10 Counts per Million (CPM)


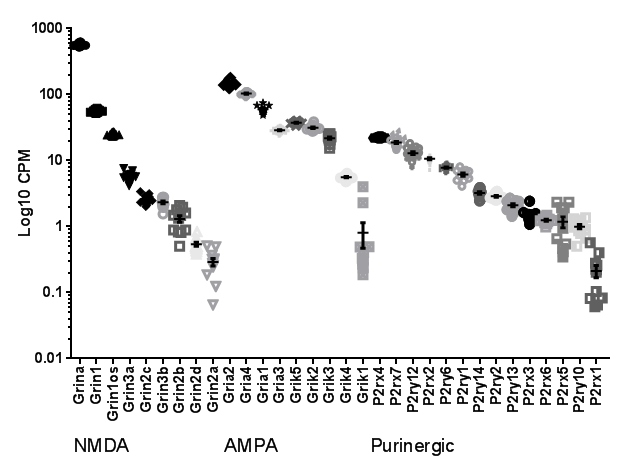


**Figure S7 continued: Receptor gene isoform expression in the stellate**

Average expression across all samples ±SEM (n=12) expressed as Log10 Counts per Million (CPM)


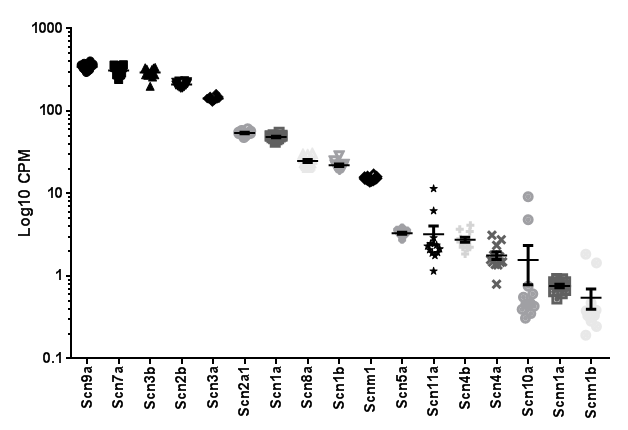


**Figure S8: Sodium channel gene isoform expression in the stellate**

Average expression across all samples ±SEM (n=12) expressed as Log10 Counts per Million (CPM)


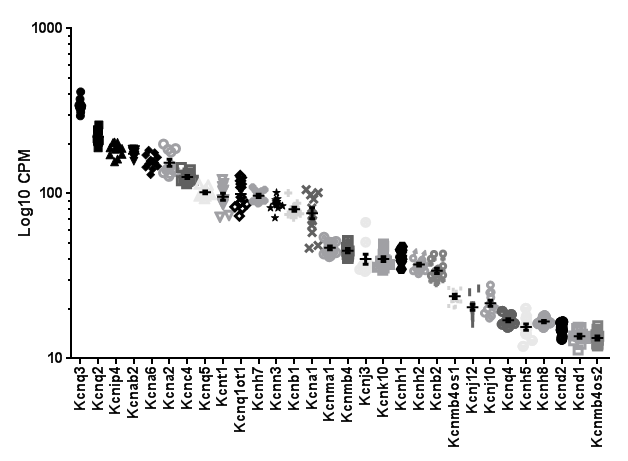


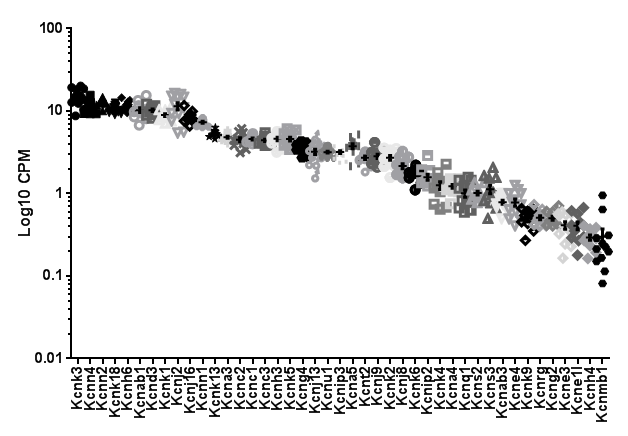


**Figure S9: Potassium channel gene isoform expression in the stellate**

Average expression across all samples ±SEM (n=12) expressed as Log10 Counts per Million (CPM)
